# A new robust Bayesian small area estimation via α‐stable model for estimating the proportion of athletic students in California

**DOI:** 10.1002/bimj.202000235

**Published:** 2021-05-07

**Authors:** Shaho Zarei, Serena Arima, Giovanna Jona Lasinio

**Affiliations:** ^1^ Department of Statistics Faculty of Science University of Kurdistan Kurdistan Iran; ^2^ Department of History, Social Science and Human Studies University of Salento Lecce Italy; ^3^ Department of Statistical Sciences University of Rome “La Sapienza” Roma Italy

**Keywords:** area‐level model, California FITNESSGRAM, hierarchical Bayesian model, small area estimation, stable distribution

## Abstract

In the last few years, diabetes mellitus and obesity revealed to be one of the fastest‐growing chronic diseases in youth in the United States. The number of new diabetes cases is dramatically increasing, and, for the moment, effective therapy does not exist. Experts believe that one of the causes of this increase is the decline in exercise behavior. The California Education Code requires local educational agencies (LEAs) to administer the FITNESSGRAM, the Physical Fitness Test (PFT), to Californian students of public schools. This test evaluates six fitness areas, and experts defined that a passing result on all six areas of the test represents a fitness level that offers some protection against the diseases associated with physical inactivity. We consider 2015–2016 data provided by the California Department of Education (CDE): for each Californian county (m=57), we aim at estimating the county‐level proportion of students with a score equal to six. To account for the heterogeneity of the phenomenon and the presence of outlying counties, we extend the standard area‐level model by specifying the random effects as a symmetric α‐stable (SαS) distribution that can accommodate different types of outlying observations. The model can accurately estimate the county‐level proportion of students with a score equal to six. Results highlight some interesting relationships with social and economic situations in each county. The performance of the proposed model is also investigated through an extensive simulation study.

## 1 INTRODUCTION

Childhood obesity continues to be one of the most significant health threats to kids and teens across developed countries. In the United States, approximately one in five youth ages 6–19 are obese, according to a recent study (Ogden et al., 2016). Childhood overweight and obesity are associated with a plethora of metabolic and clinical constraints, which result in a higher risk for the development of cardiovascular complications and metabolic disease, particularly insulin resistance and type 2 diabetes. Thus, prevention and control for childhood overweight and obesity are urgently needed. Obesity is due to a long‐term imbalance between energy intake and energy expenditure: physical activity accounts for 5–35% of total energy expenditure in children and has been regarded as a critical component in obesity prevention (Galuska et al., 2018; Metcalf et al., 2011). However, with social development and changes in people's lifestyles, children and adolescents do not spend enough time on physical activities. The national Youth Risk Behavior Surveillance (YRBS) alerted that in 2011 only 28.7% of nationwide students in the United States were physically active for a total of at least 60 min per day on each of the 7 days before the survey (Eaton et al., 2012). Therefore, the American government deems it necessary to explore effective and feasible strategies to promote childhood physical activity as the best tool for counteracting the diffusion of obesity in the USA. Schools and all educational institutes play a vital role in the dissemination of the culture of physical activities. California Education Code Section 60800 requires local educational agencies (LEAs) to administer the Physical Fitness Test (PFT) annually to students in grades five, seven, and nine. The designated PFT is the FITNESSGRAM. The test evaluates six fitness areas: Aerobic Capacity, Body Composition, Muscle Strength, Endurance and Flexibility, Trunk Extensor Strength and Flexibility, Upper Body Strength and Endurance, and Flexibility. The test is scored based on criterion‐referenced standards to evaluate fitness. A passing result in all six areas (6/6) of the test represents a fitness level that offers some protection against the diseases associated with physical inactivity (California Department of Education, 2015). In this paper, we consider data for 2015–2016 provided by the California Department of Education (CDE), and we propose a small area model for estimating the county‐level proportion of students with a score equal to six. In order to accommodate counties variability, we extend the standard area‐level model by specifying for the random effects a symmetric α‐stable (SαS) distribution that can capture different types of outlying observations.

The paper is organized as follows. In Section 2, we review the primary literature contribution about the choice of the random effect in small area models when outlying observations are involved. Methodological preliminaries about the SαS distribution are given in Section 3. The Fay–Herriot (FH) model based on SαS for the random effects is introduced in Section 4, and the computational issues related to the model estimation are outlined in Section 5. A simulation study, described in Section 6, is designed to compare the different prior distributions for the random effects in the FH model, highlighting the competitiveness of the proposed model. In Section 7, data about the proportion of athletic students in California are analyzed. The paper concludes with some discussion in Section 8.

## 2 RANDOM EFFECTS IN AREA‐LEVEL MODEL

Due to the increasing demand for small area statistics, small area estimation (SAE) is becoming one of the most interesting topics for survey statisticians. Direct design‐based estimates for small areas usually suffer from significant standard errors due to insufficient or no survey data. Consequently, model‐based estimation of small area means is receiving much attention in statistical literature where the goal is to extract information from sources other than the survey (see Molina and Rao, 2015, for a complete review).

The SAE models are mixed‐effects models involving random area‐specific effects to carry strength to the direct design‐based estimates through auxiliary information. The random effects play a crucial role in these models: the bias of model‐based small area predictors reduces by adding random effects, but, at the same time, such random effects increase the variability of the predictions, leading, in some cases, to very large confidence intervals (Fay & Graubard, 2001). Therefore, the presence or not of random effects and their specification in the model are issues in the current debate. Because of its simplicity and interpretability, the most well‐known area‐level model is the FH model (Fay & Herriot, 1979), where the random terms are assumed to be normally distributed with common variance. This assumption is very restrictive, mainly when outliers are detected in the data; hence several alternative models dealing with more flexible specifications of the random effects distribution have been proposed in the literature. In this paper, we discuss in detail the choice of random effect distribution in a Bayesian framework. We contribute to the open debate about the choice of the “optimal” prior distribution for the random effect. In Sinharay and Stern (2003), the authors explained that the distribution of the random effect significantly affects the small area estimates; however, they also showed that violations of the assumptions about the random effects are challenging to be detected using posterior‐predictive checks unless the sampling variance is small compared to the population variance. Several extensions of the FH model have been proposed in the literature to make it more robust and flexible. In Bell and Huang (2006), the authors modeled the random effects through a t distribution to deal with outliers in the data. They explained that using a t distribution with few degrees of freedom can decrease the impact of outliers on the estimation process. In Fabrizi and Trivisano (2010), the authors examine two extensions of the FH model in which the random effects are assumed to be distributed according to either an exponential power distribution (Box & Tiao, 1973) or a skewed exponential power distribution. The authors concluded that the two proposed distributions are two robust alternatives and perform adequately even when normality holds, provided that the number of areas is not too small. Although the skewed exponential power distribution is more flexible with respect to the exponential power distribution, the authors concluded that the two distributions' predictive performance is not dramatically different. They suggested that practitioners use the latter distribution since the parameters have a direct interpretation and its theoretical properties have been extensively explored. A generalized semiparametric approach has been recently proposed in Polettini (2017): the normality assumption for the random effect is replaced by a Dirichlet process that allows accommodating outlying observations. Moreover, design‐variances are hierarchically modeled according to a chi‐square distribution, embedding in the model the uncertainty on the variances that are often estimated from the same survey data.

Datta and Lahiri (1995) observed that the random effect distribution could not be the same for all areas, especially in the presence of areas where the phenomenon of interest behaves very differently from the other areas. Indeed, they propose to specify different prior distributions for different areas: for the outlying areas, they suggest using a Cauchy distribution while, for the remaining areas, they suggest an appropriate scale mixture of the normal distribution, whose tails are lighter than the Cauchy tails. However, they assume to know which areas are outlying and concluded that their method is appropriate when one or more outliers are found in the data. In Datta et al. (2011), whether or not the presence of the random effects in SAE is necessary is discussed. As already mentioned, random effects reduce the bias of the estimates but increase their variability. When the model is misspecified, and the random effects are not necessary, the predictions' variability is artificially increased with severe consequences on all inferential procedures. To check whether the random effects have to be included in the model, they introduced a test where the null hypothesis is that the common random effect variance is zero. As explained by Torkashvand et al. (2017), “Datta et al. (2011) concluded that including random effects in the model decreases the rate of convergence to the true values of area parameters.” The decrease is significant, especially when the sample size is large. They also pointed out that “dropping the random effects can lead to a more accurate point and/or interval estimators, although the flexibility and adaptivity of the area‐level (also called Fay‐Herriot) model might be lost.” Hence, this approach's main drawback is that the random effect will be omitted from all areas, whereas it might be necessary to keep it for some areas. Then, Datta and Mandal (2015) moved to a Bayesian approach and started approaching random effect inclusion as a model selection problem. They proposed to adopt a spike and slab prior distribution, defined as a mixture of a point mass at zero and a zero‐mean normal distribution. The spike component describes the probability of a particular random effect in the model to be zero, and then a regression estimate is adequate for that area; the slab component describes nonnegligible random effects when the regression model as such is not adequate. Using a similar rationale, Tang et al. (2018), introduce global–local shrinkage priors for the random effects. These priors allow area‐specific variance components for random effects. In areas where random effects are not needed, the variance component is shrunk toward zero; when random effects are needed, the variance component is larger than 0, and it increases with the role that the random effects play in each area. The proposed model is especially advantageous when the number of small areas is large, and the areas are very inhomogeneous for the phenomenon of interest. The global–local shrinkage priors employ two levels of parameters for the variances of normally distributed random effects. The first level consists of the local shrinkage parameters, which are area‐specific, while the second level involves the global shrinkage parameter specification, which is the same for all random effects. The global shrinkage parameter captures the overall/global random effect, while the local parameter captures the area‐specific random effect. The capability to model the global and local aspects of the random effects is mainly due to the prior distribution's tail. Indeed, the authors state, “If it is appropriately heavy‐tailed, both small and large random effects can be well‐captured.” However, all the aforementioned prior distributions do not account for different types of outlying observations. Atypical observations (outliers) may roughly be divided into two types: mild and gross (Ritter, 2015). A commonly used rule says that a data point is an outlier if it is more than k times the interquartile range above the third quartile or below the first quartile. An outlier is defined as mild for k∈[1.5;3) while it is gross when k≥3. Grossly atypical observations are more difficult to predict than mildly atypical observations, and random effects models are often chose to manage outlying observations, while the goodness of the predictions strongly depends on the distribution of the random effects. Indeed, the standard FH model fails in predicting gross outliers. In this work, we propose to expand FH models by replacing the Gaussian distribution there adopted with the SαS distribution that, being more flexible than the Gaussian, makes the model more robust to outliers effects.

## 3 THE α‐STABLE DISTRIBUTION

**FIGURE 1:**
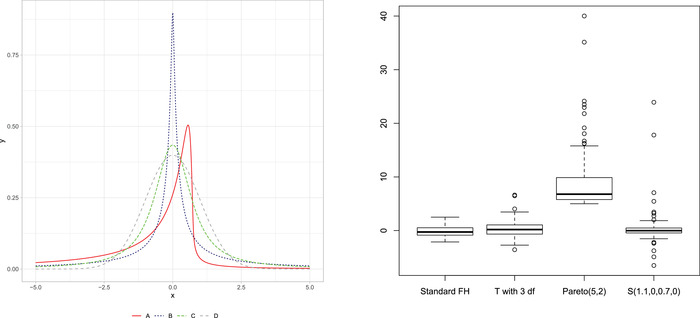
Left panel: examples of α‐stable distributions: A. α=0.5,β=−0.8,γ=1,δ=0, B. $\alpha =0.5,\beta =0,\gamma =1/\sqrt{2},\delta =0$, C. $\alpha =1.1,\beta =0,\gamma =1/\sqrt{2},\delta =0$, D. $\alpha =2,\beta =0,\gamma =1/\sqrt{2},\delta =0$. Right panel: boxplots of the data generated from different simulation scenarios

Stable (or α‐stable) distributions are a rich class of probability distributions that allow skewness and heavy tails. This class of distributions enjoys many interesting mathematical properties. Initially introduced by Lévy (1925) in his study of sums of independent identically distributed terms, they received little practical attention until the results in Mandelbrot (1961, 1963a), who introduced a simple algorithm for estimating the parameters. He referred to such distributions as “stable Paretian distributions”: in particular, he used those maximally skewed in the positive direction with 1<α<2 as “Pareto‐Lévy distributions,” which he regarded as better descriptions of stock and commodity prices than normal distributions (Mandelbrot, 1963b). The univariate α‐stable distribution is a four‐parameter family, with an index of stability (also called *tail index* or *characteristic exponent*) α∈(0,2], skewness β∈[−1,1], scale γ>0, and location δ∈R. In the following, we denote a stable random variable with aforementioned parameters as X∼S(α,β,γ,δ). The positive stable distributions have β=1 and α<1 while the SαS distributions around δ have β=0. The SαS distribution is a generalization of the Gaussian distribution having one additional parameter, α∈(0,2], governing the tails weight. The Gaussian distribution is obtained, as a special case, when α=2, while other symmetric distributions with heavier tails are obtained as α departs from 2. As an example, the Cauchy distribution is obtained for α=1. The left panel of Figure 1 shows the density of the α‐stable distribution for different parameters' different choices. For a more comprehensive discussion about the α‐stable distribution, the interested reader can refer to Nolan (2016) and Samorodnitsky and Taqqu (1994).

In general, the variance of the SαS distribution diverges to infinity when α<2 (Nolan, 2016), and this allows the models based on the SαS distribution to be more robust when gross outliers are found in the data. Moreover, SαS satisfies the generalized central limit theorem which states that the only possible nontrivial limit of normalized sums of independent identically distributed terms is stable. Given that the random effects are likely to be the sum of many small terms (maybe with a large variance), a stable model might appropriately describe such effects. The SαS distribution has scale mixtures of normals (SMiN) property. SMiN represents the symmetric stable distribution in conditionally Gaussian form. This property allows standard procedures based on the Gaussian distribution to be reused directly in statistical inferences about models with SαS terms. On the other hand, SMiN induces variances of the random effects to be expressed as the product of two levels of parameters, leading to a new type of global–local prior as introduced by Tang et al. (2018). However, since these distributions do not have a closed form for their densities functions, numerical methods must be used to employ them in modeling, and their properties are studied according to their characteristic functions. There exists many parameterizations of the stable laws characteristic functions (Nolan, 2016), the basic one being

(1) $$\varphi \left(w;\alpha ,\beta ,\gamma ,\delta \right)=\exp\left(iw\delta -{\left|\gamma w\right|}^{\alpha }\left(1-i\beta \mathrm{sign}(w)\Phi \right)\right),$$

where sign(.) is the sign function and

$$\Phi =\left\{\begin{array}{cc} \tan\left(\frac{\pi \alpha }{2}\right) & \alpha \neq 1\\ -\frac{2}{\pi }\log\left|w\right| & \alpha =1. \end{array}\right.$$

However, (1) shows discontinuities in the parameters when α=1. In Nolan (2016), a simple form for the characteristic function of the SαS distribution is derived as well as some useful algebraic properties. In particular, they introduced the 1‐parameterization defined as

(2) $$\phi \left(w\right)=\left\{\begin{array}{cc} e^{-{\left|w\gamma \right|}^{\alpha }\left[1-i\mathrm{sign}\left(w\right)\beta \tan\left(\frac{\pi \alpha }{2}\right)\right]+i\mu w} & \alpha \neq 1,\\ e^{-\left|w\gamma \right|\left[1+i\frac{2}{\pi }\mathrm{sign}\left(w\right)\beta \log\left(|w|\right)\right]+i\mu w} & \alpha =1. \end{array}\right.$$

From Equation (2), it can be easily shown that $S(2,0,\frac{\gamma }{\sqrt{2}},\mu )$ is the Gaussian distribution with mean μ and variance $\gamma^{2}$.

## 4 BASIC AREA‐LEVEL MODEL WITH SαS RANDOM EFFECTS

The FH model (Fay & Herriot, 1979) is defined as follows:

(3) $$y_{i}=x_{i}^{T}\beta +v_{i}+e_{i},\;\;\;\;\;\;\;\;i=1,\cdots ,m,$$

where $y_{i}$ is the direct estimate for small area i, $x_{i}$ is the p‐dimensional vector of covariates, $\beta ={\left(\beta_{1}\cdots ,\beta_{k}\right)}^{T}$ is a p‐dimensional vector of regression coefficients, and m is the number of small areas. The error vector $e={\left(e_{1},\cdots ,e_{m}\right)}^{T}$ and the random effect vector $v={\left(v_{1},\cdots ,v_{m}\right)}^{T}$ are assumed to be independent. The elements of e are independent with $e_{i}∼N\left(0,\psi_{i}\right)$, where $\psi_{i}$s are typically assumed to be known. When only estimates of the sampling variance are available, a model shrinking both means and variances should be considered. In particular, Maiti et al. (2014) noticed that when the sampling variances are estimated quantities, these are subject to substantial errors because they are often based on equivalent sample sizes as the direct estimates are being calculated. Indeed, they proposed to extend the FH model in order to account the uncertainty of (sampling variance) estimation into the overall SAE strategy. They estimate the model parameters according to an empirical Bayes approach. This idea has been then extended by Sugasawa et al. (2017), who propose a Bayesian hierarchical model for the sample variance and discuss different prior choices.

With respect to the area effects, the FH model assumes that $v_{i}$s are independent and identically distributed (i.i.d) random variables with $N(0,\sigma_{v}^{2})$. Such an assumption makes the FH model not suitable in the presence of outliers. We proposed a robustification of the FH model for the presence of atypical observations by modeling the random effects $v_{i}$ with the SαS distribution. In the following, the proposed model will be denoted as Fay‐Herriot α‐stable (FHαS) model. The SαS distribution allows capturing outlying observation since over the commonly used robustified small area models. The proposed FHαS model is as follows:

(4) $$y_{i}=x_{i}^{T}\beta +v_{i}+e_{i},\;\;\;\;\;\;\;\;i=1,\cdots ,m,$$

where $v_{i}\overset{i.i.d}{∼}S\left(\alpha ,0,\gamma_{1},0\right)$ and $e_{i}\overset{ind.}{∼}N\left(0,\psi_{i}\right)$. The ultimate goal of the proposed model is that it predicts the small area parameter (mean) $\theta_{i}=x_{i}^{T}\beta +v_{i}$ with a higher precision than competing approaches.

Following Molina and Rao (2015), the proposed model can be written in the following Bayesian hierarchical way:

- $y_{i}|v_{i},\beta ,\gamma_{1},\alpha ∼N\left(x_{i}^{T}\beta +v_{i},\psi_{i}\right)$,
- $v_{i}|\beta ,\gamma_{1},\alpha ∼S\left(\alpha ,0,\gamma_{1},0\right)$,
- $\beta ∼N(\mu_{\beta },\Sigma_{\beta })$; $\pi \left(\alpha \right)\propto \frac{1}{2}$; $\gamma_{1}∼\pi_{1}\left(.\right)$,

where $\pi_{1}\left(.\right)$ is a density function with positive support adopted as prior distribution for $\gamma_{1}$.

For the regression parameters β, we assume “flat” prior by specifying a multivariate normal distribution with large variances. Without loosing of generality, we assume $\mu_{\beta }=0$ and $\Sigma_{\beta }=\mathrm{diag}\left(10^{5}\right)$. Adopting the same approach as Salas‐Gonzalez et al. (2010), Lombardi (2007), and Tsionas (1999), we choose a Uniform distribution in the interval (0,2] for the tail index α. Since the closed form of $S(\alpha ,0,\gamma_{1},0)$ is not available, the model as it stands, is hard to be estimated: the joint posterior distribution cannot be computed analytically but also the derivation of full conditional distributions is a complex task. From a computational point of view, it is then convenient to reparameterize the model using the SMiN property of SαS distribution (Samorodnitsky & Taqqu, 1994), more precisely:

Definition 1 Suppose Z is a zero‐mean Gaussian random variable with variance $\sigma^{2}$ and $P∼S(\frac{\alpha }{2},1,{\left(\cos\left(\frac{\pi \alpha }{4}\right)\right)}^{\frac{2}{\alpha }},0)$ is a positive stable random variable, independent of Z then the random variable $X=\mu +\sqrt{P}Z$ is S($\alpha ,0,\frac{\sigma }{\sqrt{2}},\mu$), where μ is a constant.

According to Definition 1, the model can be reparameterized as follows:

- $y_{i}|v_{i},\beta ,\gamma_{1},\alpha ∼N\left(x_{i}^{T}\beta +v_{i},\psi_{i}\right)$,
- $v_{i}|\lambda_{i},\beta ,\gamma_{1},\alpha ∼N\left(0,\lambda_{i}\gamma \right)$; $\beta ∼N(\mu_{\beta },\Sigma_{\beta })$,
- $\lambda_{i}|\alpha ∼S\left(\frac{\alpha }{2},1,{\left(\cos\left(\frac{\alpha \pi }{2}\right)\right)}^{\frac{2}{\alpha }},0\right)$; $\pi \left(\alpha \right)\propto \frac{1}{2}$; γ∼IG(a,b),

where $\gamma =2\gamma_{1}^{2}$ and IG(a,b) denote inverse Gamma distribution with known shape and scale parameters a and b, respectively. Notice that this structure of the random effects is a particular type of the global–local shrinkage prior for the random effects (Tang et al., 2018). The variance parameters of the random effects are area‐specific. For each small area, they are expressed as the product of a global parameter γ and a local parameter $\lambda_{i}$: the former captures the overall random effects while the latter brings extra variability to the random effects compared to the FH model. According to the Bayes' theorem, the posterior distribution is given by

(5) π(v,β,γ,α,λ|y)=π(y|v,β,γ,α,λ)π(v|β,γ,α,λ)π(λ|α)π(α)π(β)π(γ),

where $y={\left(y_{1},\cdots ,y_{m}\right)}^{T}$ is the observations vector and $\lambda ={\left(\lambda_{1},\cdots ,\lambda_{m}\right)}^{T}$ is the vector of latent local random effects. Given the complexity of the posterior distribution (5), numerical methods, and Monte Carlo Markov Chain (MCMC) algorithm should be applied in order to sample from it. In particular, we use a combination of the Gibbs sampling, rejection sampling, and Metropolis–Hastings algorithm. Our proposal is developed under a fully Bayesian approach.

## 5 COMPUTATIONAL DETAILS

The estimation of the proposed model is tricky and cannot be rephrased in the standard MCMC framework. It requires the application of different sampling algorithms to sample efficiently from the joint posterior distribution. In particular, Gibbs sampling can be involved for β, γ, and $v_{i}$ (i=1,⋯,m), since full conditional distributions can be derived in closed form. For the other parameters, λ and α, rejection sampling and Metropolis–Hastings algorithm will be used.

### 5.1 Updating β, v, and γ

Parameters β, v, and γ are updated through Gibbs sampling algorithm according to the following full conditional distributions:

1. $\pi \left(\beta |v,\gamma ,\alpha ,\lambda ,y\right)∼N({\left(X\Sigma^{-1}X^{T}+\Sigma_{\beta }^{-1}\right)}^{-1}\left(X\Sigma^{-1}\left(y-v\right)\right),\;{\left(X\Sigma^{-1}X^{T}+\Sigma_{\beta }^{-1}\right)}^{-1})$
2. $\pi \left(v_{i}|\beta ,\gamma ,\alpha ,\lambda ,y\right)∼N\left(\delta_{i}\left(y_{i}-x_{i}^{T}\beta \right),\;\delta_{i}\psi_{i}\right)$
3. $\pi \left(\gamma^{-1}|v,\alpha ,\lambda ,\beta ,y\right)∼G\left(\frac{m}{2}+a,\;\sum_{i}\frac{v_{i}^{2}}{2\lambda_{i}}+b\right)$,

where $\Sigma =\mathrm{diag}(\psi_{1},\cdots ,\psi_{m})\;$ and $\delta_{i}=\frac{\lambda_{i}\gamma }{\lambda_{i}\gamma +\psi_{i}}$ for i=1,⋯,m.

### 5.2 Updating the vector of local parameters λ

The posterior distribution of λ is defined as

π(λ|v,γ,α,β,y)∝π(v|β,γ,α,λ)π(λ|α).

Since π(λ|α) does not have a closed form, we use rejection sampling for updating $\lambda_{i}$ (i=1,⋯,m). Following Godsill and Kuruoglu (1999) and Zarei and Mohammdpour (2020), the posterior distribution can be expressed as

$$\pi \left(\lambda |v,\gamma ,\alpha ,\beta ,y\right)=\prod_{i=1}^{m}\pi \left(\lambda_{i}|v_{i},\gamma ,\alpha ,\beta ,y_{i}\right)\propto \prod_{i=1}^{m}N\left(v_{i}|0,\lambda_{i}\gamma \right)f_{\frac{\alpha }{2}}\left(\lambda_{i}\right),$$

where $N(v_{i}|0,\lambda_{i}\gamma )$ denotes the probability density function of a normal distribution with zero mean and variance equals to $\lambda_{i}\gamma$ and $f_{\frac{\alpha }{2}}\left(.\right)$ is the density function of the positive stable random variable. It is readily seen that the likelihood forms a valid rejection function as it is bounded from above:

$$N\left(v_{i}|0,\lambda_{i}\gamma \right)\leq \frac{1}{\sqrt{2\pi v_{i}^{2}}}\exp\left(-\frac{1}{2}\right).$$

A suitable rejection sampler is:

1. Draw a sample, $\lambda_{i}^{*}$, from the positive stable distribution with the tail index α/2.
2. Draw a sample, u, from the $U(0,\frac{1}{\sqrt{2\pi v_{i}^{2}}}\exp\left(\frac{-1}{2}\right))$.
3. If $u<N(v_{i}|0,\lambda_{i}\gamma )$ accept $\lambda_{i}^{*}$, otherwise go to 1.

### 5.3 Updating the tail index α

Since π(α|v,γ,λ,β,y)∝π(λ|α)π(α) and $\pi \left(\lambda |\alpha \right)=\prod_{i=1}^{m}\pi \left(\lambda_{i}|\alpha \right)$ does not have a closed form, the parameter α is estimated using the Metropolis–Hastings algorithm. For each $\lambda_{i}$, we choose the Uniform distribution centered on the current state of the chain, denoted by *cand*, as proposal distribution, that is, $q\left(\lambda_{i}|\alpha \right)∼U\left(cand-0.15,cand+0.15\right)$. Since the Uniform distribution is symmetric, the acceptance probability in the each iteration is

(6) $$\min\left\{1,\frac{\prod_{i=1}^{m}\pi \left(\lambda_{i}|\alpha^{new}\right)}{\prod_{i=1}^{m}\pi \left(\lambda_{i}|\alpha^{\left(t\right)}\right)}\right\}.$$

## 6 SIMULATION

In this section, we compare the proposed model's performance with the direct estimates, the standard FH model, and the following competing models:

1. The model proposed in Datta and Mandal (2015) (hereafter denoted as DM model). In such a model, the random effects are modeled with spike‐and‐slab distributions, that is, a mixture of a point mass at zero and a zero‐mean normal distribution;
2. The models proposed in Tang et al. (2018), where the random effects follow a global–local prior distribution. We consider two possible specifications of global and local parameters: (A) The global parameter is modeled as Inverse Gamma, and prior distributions for the local parameters set to be normal‐Gamma (hereafter denoted as GL‐NG). (B) The global parameter is modeled as Inverse Gamma and Laplace distribution (hereafter denoted as GL‐LA) as prior distributions for the local parameters.
3. The model in which the random effect follows a Student's t distribution with k degrees of freedom, $v_{i}∼t_{k}$ and k is modeled as a discrete uniform distribution ranging in the interval 0–50 (hereafter denoted as t model);
4. The model proposed in Fabrizi and Trivisano (2010), where the random effects follow an exponential power distribution (hereafter denoted EP).

The data generation settings are adopted from Chakraborty et al. (2016) and Tang et al. (2018). For comparability with the real data applications, the number of small areas, m, is set to 20, 50, and 100. For each choice of m, we generated data from the model (3) with different specifications of the random effects allowing for different outlying behavior of the observed data. Scenario 1 is the standard FH model with normally distributed random effects, $v_{i}∼N\left(0,1\right)$. More weight to values on the tails is given in Scenario 2, where the random effects are distributed as a Student t distribution with 3 degrees of freedom, $v_{i}∼t_{3}$ (here after t model). In this setting, Tang et al. (2018) showed that the best fitting model is those with global–local prior for the random effects (GL‐LA or GL‐NG). In Scenario 3, the model generating the data is the proposed model, $v_{i}∼S\left(1.1,0,\frac{1}{\sqrt{2}},0\right)$. To investigate the performance of the models when data are generated from a probability distribution that cannot be reconnected to one of the distributions mentioned above, in Scenario 4, we generate data from an FH model with random effects distributed as a Pareto distribution with scale and shape parameters, respectively, equal to 5 and 2, $v_{i}∼Pareto\left(5,2\right)$. Figure 1, right panel, and Figure 1 in the Supplementary Materials present the data's effect when random effects are distributed according to the scenarios above. They show that the data distribution is strongly affected by the distribution of the random effects. When random effects are generated from $t_{3}$, the outliers are mild, while when the random effects are generated according to Scenario 3 and 4, the resulting data are skewed with the gross outlying observations. With the Pareto distribution in Scenario 4, we allow the generation of very large values for the random effects, occasionally inducing very large outliers. The design matrix X includes a column of ones and one explanatory variable sampled from N(10,2). The coefficient vector β is fixed at ${\left(20,1\right)}^{T}$. Errors' variances, $\psi_{i}$, are chosen from the set {0,0.5,1,1.5,2,2.5,3,3.5,4,4.5,5}, and each value in the set is allocated to the same number of small areas. The hyperparameters of all prior distributions are set to be noninformative. In Scenario 3, we fix α=1.1.

Performance in estimating small area means $\theta_{i}=x_{i}^{T}\beta +v_{i}$ are studied according to the following deviance measures: average absolute deviation (AAD), average squared deviation (ASD), average absolute relative deviation (ARB), average squared relative deviation (ASRB), defined as follows:

$$\begin{array}{ccc} AAD & = & \frac{1}{m}\sum_{i=1}^{m}\left|{\hat{\theta }}_{i}-\theta_{i}\right|,\;\;\;\;\;\;ASD=\frac{1}{m}\sum_{i=1}^{m}{\left({\hat{\theta }}_{i}-\theta_{i}\right)}^{2},\\ ARB & = & \frac{1}{m}\sum_{i=1}^{m}\left|\left({\hat{\theta }}_{i}-\theta_{i}\right)/\theta_{i}\right|,\;\;ASRB=\frac{1}{m}\sum_{i=1}^{m}{\left({\hat{\theta }}_{i}-\theta_{i}\right)}^{2}/\theta_{i}^{2}. \end{array}$$

We also consider the empirical coverage rate (hereafter CR) of 95% credible interval of $\theta_{i}$. In practice, it is not possible to know the underlying true model, and the true value of $\theta_{i}$ is unknown, so how to choose an appropriate prior for the random effects based on the data is an important task. We use deviance information criterion (DIC; Spiegelhalter et al., 2002) for evaluation of goodness of fit. The following tables show the results of simulations in different scenarios, averaged over the 50 data sets.

Table 1 shows the deviance measures when the random effects are generated under Scenario 1, 2, and 3. As expected, the FH model has the best overall performance; moreover, DM and FHαS perform very similarly, and the deviance measures are not substantially different from those of the FH model. That is not a surprising result since DM model essentially is based on the normal distribution, and FHαS covers the normal distribution as a special case (α=2). Notice that, especially when the number of areas is small (m=20), the Student t model performs similarly and, in some cases, even better than the FH model. That is in agreement with the conclusions in Fabrizi and Trivisano (2010).

**TABLE 1:** Models performances according to average absolute deviation (AAD), average squared deviation (ASD), average absolute relative deviation (ARB), average squared relative deviation (ASRB), deviance information criterion (DIC) and coverage rate (CR)

			m					m		
	Criteria	Estimate	20	50	100	Criteria	Estimate	20	50	100
**Scenario 1**										
		FHαS	0.799	0.708	0.705		FHαS	1.032	0.818	0.802
		FH	0.780	**0.690**	**0.701**		FH	0.972	**0.768**	**0.783**
	ADD	DM	0.764	0.702	0.714	ASD	DM	0.925	0.795	0.818
		GL‐NG	0.791	0.720	0.723		GL‐NG	1.002	0.838	0.848
		GL‐LA	0.795	0.714	0.717		GL‐LA	1.001	0.826	0.829
		T	**0.741**	0.691	0.781		T	**0.883**	0.799	0.731
		EP	1.07	1.039	0.990		EP	1.864	1.743	1.624
		Direct	1.270	1.275	1.270		Direct	2.759	2.768	2.747
		FHαS	0.0268	0.0239	0.0237		FHαS	0.0012	0.0009	0.0009
		FH	0.0262	**0.0231**	**0.0234**		FH	0.0011	0.0009	0.0009
	ARB	DM	**0.0255**	0.0235	0.0239	ASRB	DM	**0.0010**	0.0009	0.0009
		GL‐NG	0.0266	0.0242	0.0243		GL‐NG	0.0011	0.0009	0.0010
		GL‐LA	0.0266	0.0240	0.0239		GL‐LA	0.0011	0.0009	0.0009
		T	0.0256	0.0232	0.0288		T	0.0010	0.0009	0.0008
		EP	0.0362	0.0349	0.0331		EP	0.0002	0.0006	0.0003
		Direct	0.0428	0.0427	0.0426		Direct	0.0032	0.0031	0.0031
		FHαS	**81.67**	**201.54**	400.54		FHαS	**0.97**	**0.96**	**0.94**
		FH	81.68	201.82	**400.17**		FH	**0.97**	0.95	**0.94**
	DIC	DM	82.17	202.62	403.45	CR	DM	0.88	0.87	0.90
		GL‐NG	82.97	204.05	403.89		GL‐NG	0.82	0.81	0.84
		GL‐LA	82.72	203.38	402.85		GL‐LA	0.82	0.83	0.88
		T	81.74	201.83	402.30		T	0.95	0.96	0.98
		EP	87.684	216.827	433.32		EP	0.90	0.98	0.96
**Scenario 2**										
	Criteria	Estimate	20	50	100	Criteria	Estimate	20	50	100
		FHαS	0.923	0.876	0.826		FHαS	1.473	1.289	1.165
		FH	0.929	0.911	0.875		FH	1.517	1.431	1.359
	ADD	DM	0.945	0.875	0.839	ASD	DM	1.597	1.354	1.245
		GL‐NG	0.978	0.870	0.825		GL‐NG	1.687	1.289	1.182
		GL‐LA	0.985	0.874	0.827		GL‐LA	1.715	1.304	1.188
		T	**0.891**	**0.841**	**0.801**		T	**1.416**	**1.233**	**1.127**
		EP	1.075	1.074	1.025		EP	1.969	1.934	1.729
		Direct	1.254	1.329	1.267		Direct	2.684	2.958	2.753
		FHαS	**0.0312**	0.0296	**0.0278**		FHαS	0.0017	0.0015	**0.0014**
		FH	0.0313	0.0307	0.0300		FH	0.0017	0.0018	0.0030
	ARB	DM	0.0318	0.0295	0.0284	ASRB	DM	0.0018	0.0017	0.0015
		GL‐NG	0.0329	0.0293	0.0278		GL‐NG	0.0019	0.0015	0.0014
		GL‐LA	0.0332	0.0295	0.0280		GL‐LA	0.0020	0.0016	0.0014
		T	0.0401	**0.0280**	**0.0270**		T	**0.0010**	**0.0014**	**0.0013**
		EP	0.0363	0.0363	0.0346		EP	0.0010	0.0080	0.0030
		Direct	0.0421	0.0446	0.0425		Direct	0.0030	0.0034	0.0031
		FHαS	83.93	**209.78**	**414.89**		FHαS	0.95	0.95	0.94
		FH	84.46	212.35	421.29		FH	0.94	0.95	0.93
	DIC	DM	85.10	211.44	419.54	CR	DM	0.84	0.88	0.89
		GL‐NG	85.61	209.96	415.85		GL‐NG	0.80	0.91	0.93
		GL‐LA	85.54	210.47	415.50		GL‐LA	0.80	0.92	0.94
		T	**83.14**	209.87	415.32		T	**0.99**	**0.96**	**0.97**
		EP	88.415	219.64	437.77		EP	**0.99**	0.94	0.94
**Scenario 3**										
	Criteria	Estimate	20	50	100	Criteria	Estimate	20	50	100
		FH1.1S	0.956	**0.887**	**0.843**		FH1.1S	1.663	1.451	**1.346**
		FHαS	**0.933**	0.888	0.846		FHαS	**1.507**	**1.424**	1.367
		FH	1.091	1.082	1.190		FH	2.094	2.056	2.488
	ADD	DM	0.962	0.918	0.889	ASD	DM	1.726	1.653	1.587
		GL‐NG	0.976	0.921	0.927		GL‐NG	1.745	1.510	1.571
		GL‐LA	1.012	0.965	0.997		GL‐LA	1.866	1.643	1.764
		T	0.965	0.910	0.876		T	1.582	1.524	1.433
		EP	1.159	1.146	1.102		EP	2.272	2.220	2.061
		Direct	1.268	1.248	1.281		Direct	2.785	2.640	2.840
		FH1.1S	0.0330	**0.0301**	0.0324		FH1.1S	**0.0023**	**0.0018**	**0.0023**
		FHαS	**0.0326**	0.0310	**0.0294**		FHαS	0.0026	0.0030	0.0024
		FH	0.0382	0.0370	0.0446		FH	0.0037	0.0029	0.1403
	ARB	DM	0.0336	0.0314	0.0330	ASRB	DM	0.0032	0.0025	0.0075
		GL‐NG	0.0341	0.0315	0.0326		GL‐NG	0.0029	0.0023	0.0039
		GL‐LA	0.0354	0.0329	0.0352		GL‐LA	0.0032	0.0024	0.0065
		T	0.0332	0.0320	0.0285		T	0.0017	0.0332	0.0098
		EP	0.0388	0.0414	0.0373		EP	0.0015	0.0002	0.0002
		Direct	0.0434	0.0425	0.0455		Direct	0.0036	0.0042	0.0206
		FH1.1S	85.50	**209.17**	**419.80**		FH1.1S	0.95	0.95	0.95
		FHαS	**85.19**	212.32	422.77		FHαS	**0.96**	**0.96**	0.95
		FH	88.93	223.17	456.87		FH	0.95	0.95	0.94
	DIC	DM	86.94	214.88	438.31	CR	DM	0.85	0.78	0.60
		GL‐NG	86.62	212.91	427.44		GL‐NG	0.93	**0.96**	**0.97**
		GL‐LA	87.70	215.92	433.65		GL‐LA	0.92	**0.96**	0.96
		T	85.39	210.97	442.15		T	0.90	0.96	0.96
		EP	89.45	223.046	447.00		EP	0.96	0.96	0.96

*Note: All performance diagnostics are reported under Scenario 1, 2, and 3 as described in Section *6. *Bold numbers highlight the best performance for each setting*.

In t‐student setting, the deviance measures show that the best fitting model is, as expected, the t model. However, when the number of small area increases, the GL‐NG model and the FHαS perform very similarly to the model generating the data. When the data are generated from a model with random effects distributed according to the SαS distribution, results confirm that the proposed model outperforms the competing models. Results also highlight that when m is small, the DM model performs better than the other models, while the GL‐NG model performs better for large m. Also, in this case, the t model performs well when the number of small areas is small. To further investigate the performance of FHαS in Scenario 3, we add a comparison with an SαS model in which α is known (FH1.1S): whether the tail index is known or unknown, the proposed model behaves better than the others, as confirmed by the results in Table 1. That means that the tail index is accurately estimated, and its knowledge leads only to a slight improvement of the estimates.

When the data are generated from a model that cannot be reconnected to one of the models under comparison, Scenario 4, the proposed model's robustness to the competing ones emerges. Indeed, Table 2 shows that for all values of m, FHαS has the best overall performance according to deviance measures and DIC, followed by GL models. This result is quite expected since when data are generated from a Pareto distribution, we faced gross and mild outliers that can be flexibly accommodated by the SαS distribution.

**TABLE 2:** Comparison of different models performances when data are simulated under Scenario 4, $v_{i}∼\mathrm{Pareto}\left(5,2\right)$

		m					m		
Criteria	Estimate	20	50	100	Criteria	Estimate	20	50	100
	FHαS	**1.114**	**1.071**	**1.064**		FHαS	**2.196**	**1.998**	**1.958**
	FH	1.202	1.211	1.232		FH	2.574	2.482	2.542
ADD	DM	1.202	1.118	1.135	ASD	DM	2.523	2.176	2.242
	GL‐NG	1.124	1.091	1.088		GL‐NG	2.243	2.033	2.016
	GL‐LA	1.150	1.125	1.127		GL‐LA	2.348	2.152	2.132
	T	1.197	1.095	1.113		T	2.278	2.053	2.135
	ExpPow	1.198	1.209	1.205		ExpPow	2.426	2.509	2.454
	Direct	1.242	1.262	1.271		Direct	2.726	2.716	2.697
	FHαS	**0.0284**	**0.0272**	**0.0271**		FHαS	**0.0014**	**0.0012**	**0.0012**
	FH	0.0307	0.0310	0.0317		FH	0.0017	0.0016	0.0016
ARB	DM	0.0304	0.0283	0.0289	ASRB	DM	0.0016	0.0014	0.0014
	GL‐NG	0.0286	0.0278	0.0279		GL‐NG	**0.0014**	0.0013	0.0013
	GL‐LA	0.0293	0.0287	0.0289		GL‐LA	0.0015	0.0014	0.0014
	T	0.0290	0.0285	0.0280		T	0.0012	0.0012	0.0013
	ExpPow	0.0300	0.0276	0.0310		ExpPow	0.0016	0.0013	0.0013
	Direct	0.0321	0.0326	0.0331		Direct	0.0018	0.0018	0.0018
	FHαS	**89.66**	**220.77**	**437.32**		FHαS	0.95	**0.95**	0.94
	FH	91.48	229.73	460.02		FH	0.95	0.94	**0.95**
DIC	DM	92.43	227.37	451.97	CR	DM	0.87	0.78	0.74
	GL‐NG	90.40	222.34	441.12		GL‐NG	0.95	**0.95**	**0.95**
	GL‐LA	90.56	224.18	445.22		GL‐LA	**0.96**	**0.95**	**0.95**
	T	90.62	225.82	444.778		T	0.94	0.95	0.92
	ExpPow	91.12	227.51	454.270		ExpPow	0.90	0.94	**0.95**

Note: Model performances are measured according to average absolute deviation (AAD), average squared deviation (ASD), average absolute relative deviation (ARB), average squared relative deviation (ASRB), deviance information criterion (DIC), and coverage rate (CR).

## 7 REAL DATA ANALYSIS: ESTIMATING THE PROPORTION OF ATHLETIC STUDENTS IN CALIFORNIA

In the last decades, diabetes mellitus and obesity revealed to be among the most common chronic diseases in youth in the United States. The number of new diabetes cases among youth increased from 3% type 2 cases in 1990 to 45% type 2 cases in 2005. Experts ascribed this significant change to the increase in obesity and a decline in exercise behaviors (Price et al., 2013). This phenomenon dramatically affected the United Stated, but it is particularly severe in California, where 38.8% of low‐income children and adolescents aged 2–19 years, in 2010, were overweight or obese. To study in more detail the diffusion of this phenomenon and determine more specialized political actions, California Education Code Section 60800 requires LEAs to administer the FITNESSGRAM, a PFT, to Californian students. The FITNESSGRAM is a comprehensive, health‐related physical fitness battery developed by The Cooper Institute, whose primary goal is to help students establish lifetime habits of regular physical activity. Public school students in grades five, seven, and nine are required to take the PFT, whether or not they are enrolled in a physical education class or participate in a block schedule. These students include those enrolled in LEAs such as elementary, high, and unified school districts, county offices of education, and charter schools. LEAs must also test all students in alternate programs, including, but not limited to, continuation schools, independent study, community day schools, county community schools, and nonpublic schools. The test evaluates six fitness areas: Aerobic Capacity, Body Composition, Muscle Strength, Endurance and Flexibility, Trunk Extensor Strength and Flexibility, Upper Body Strength and Endurance, and Flexibility. The test is scored based on criterion‐referenced standards to evaluate fitness. Experts defined that a passing result on all six areas of the test represents a fitness level that offers some protection against the diseases associated with physical inactivity (California Department of Education, 2015). We consider 2015–2016 data provided by the CDE (http://www.cde.ca.gov/ta/tg/pf/pftresearch.asp). The survey is based on a stratified sample where strata correspond to three grades (5th, 7th, 9th), seven race/ethnicity groups (AIAN, AfricanAm, Asian, Latino, Multiple, NHOPI, White), three school types (all schools, noncharter, charter). The direct estimates $y_{i}$, that is, the proportion of children that obtain a result 6/6 on the FITNESSGRAM and the design‐based variance estimate $\psi_{i}$ are directly available on the CDE website. The direct estimators are Horvitz–Thompson means, and the design‐based variances have been computed with Taylor linearization approximations. We refer to the CDE website for more details about the sampling procedure.

The sample size (m=57) and the proportion of athletic students allow us to use a Gaussian model, although $y_{i}$ is the mean of binary variables indicating whether a sampled student shows a score equal to six. The $y_{i}$s range from 18.18 to 57.36 with a median of 31.14, while standard errors range from 2.24 to 9.6 with a median of 5.374.

As a second data source, we consider the 2015 ACS data available on the Census web page. In particular, for each county in the California state, we collect the following information: percent of under 18 population, percent of the Hispanic population, percent of the White population, percent of the Black population, percent of the population with Bachelor or higher school degree, the median income, and the percent of persons in poverty . All these variables have been used as covariates in the small area model. We also considered many other variables related to the county's urban/rural status and various characteristics of the population, such as percent of the population with a disability and the mean time spent for reaching the workplace. They did not contribute much when adjusting for the other seven covariates included in the model.

**FIGURE 2:**
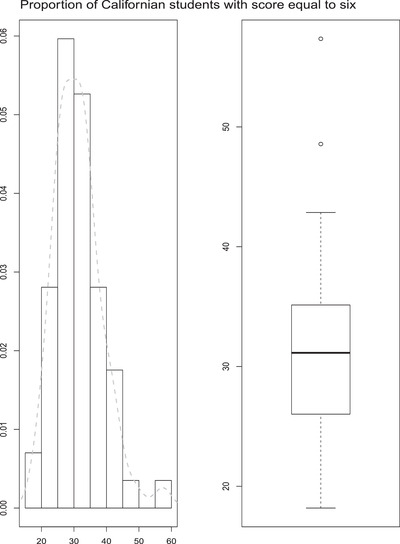
PFT data: histogram on left panel and boxplot on the right panel of the proportion of Californian students with a score equal to six

Figure 2 shows the histogram and the direct estimator's boxplot, that is, the proportion of Californian students with a score equal to six. The distribution seems to be quite asymmetric, with few potential outlying observations suggesting a more flexible model like the one proposed in the previous sections.

**FIGURE 3:**
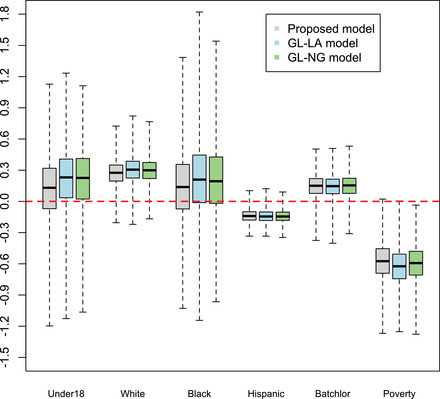
Posterior distribution of the model parameters: proposed model and competing models

**FIGURE 4:**
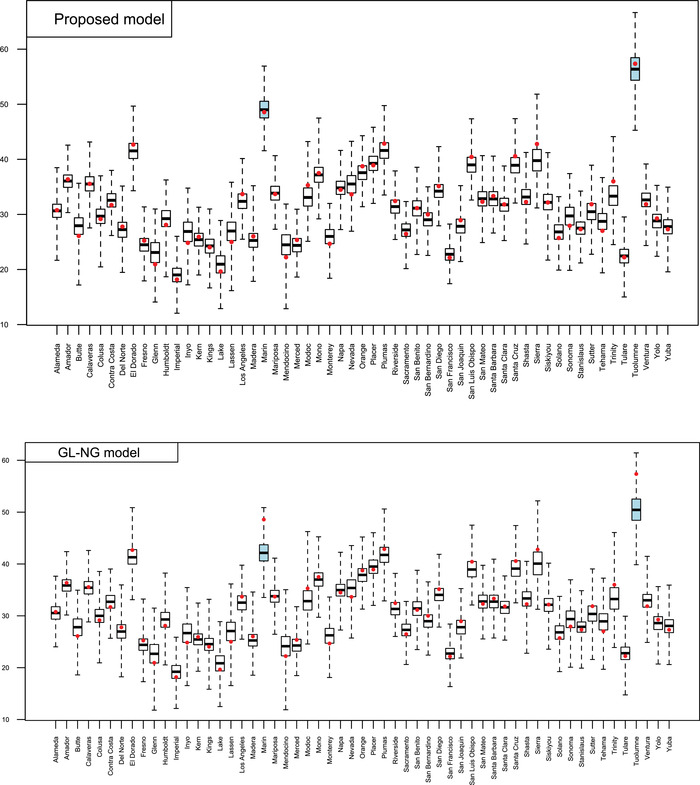
Posterior distribution of the small area means of the proposed model (upper panel) and the GL‐NG model (lower panel). Red points correspond to the direct estimates

The proposed model has been compared to the aforementioned competing model: the DIC of the proposed model resulted, respectively, equal to 303.35, while for the FH, DM, GL‐NG, and GL‐LA resulted, respectively, equal to 305.00, 306.69, 303.78, and 303.84. The proposed model fits the data better than other models, followed by the Normal Gamma (GL‐NG) model and the GL‐LA. This result agrees with the simulation study according to which, when data show largely outlying observations, like those generated (see Table 2), the proposed model, GL‐NG, and GL‐LA models perform very similarly, especially when the number of areas is moderate. In Figure 3, we report the estimates of the model parameters for the proposed model and the two most competing models. According to all models, students' proportion with a score equal to six is positively influenced by the percentage of the White population and the percentage of persons with a high cultural level in the county. On the other hand, such a proportion is negatively influenced by the Hispanic population's percentage and the poverty level. These results agree with the current literature asserting that youth of color and those of low socioeconomic status are disproportionally impacted by diabetes and obesity (Price et al., 2013). Figure 4 shows the small area mean's posterior distribution for the proposed model and the GL‐NG model. A very similar plot can be done considering the GL‐LA model. The figure highlights the presence of two counties, Marin and Tuolumne show an outlying proportion of students with a score equal to six. Marin's county is well known to be one of the richest counties in the United States, showing the highest pro‐capita mean income. Toulomne county has one of the most recognized and well‐administered scholastic systems in the USA. Indeed, schools in that county have an average ranking in the 7 over 10, having one of the highest concentrations of top‐ranked public schools in California. The proposed model correctly identifies these two outlying counties while the competing model underestimates them.

In order to investigate the robustness of the model with respect to the presence of the outlying counties, following Arima et al. (2014), we performed a leave‐one‐out cross‐validation: we computed the cross‐validation error according to the following formula: $CV=\frac{1}{m}\sum_{i=1}^{m}{\left({\hat{\theta }}_{i}-{\hat{\theta }}_{i}^{\left(-1\right)}\right)}^{2}$, where ${\hat{\theta }}_{i}$ is the posterior small area mean of the ith area and ${\hat{\theta }}_{i}^{\left(-1\right)}$ is the posterior small area mean when the ith observation in removed. Figure 3 in the Supplementary Materials shows the small area's posterior distribution means for the proposed model (light blue boxes) and for the proposed model fitted removing the two outlying counties (gray boxes). No significant differences might be grasped. Indeed, when the two counties are removed, the cross‐validation error is equal to 0.02, confirming the robustness of the proposed model.

## 8 CONCLUSIONS

In this paper, we present an extension of the FH model based on the SαS distribution (FHαS). We act in a fully Bayesian framework to handle large outliers inducing high variability in the random effects. Our proposal can be stated as a hierarchical Bayesian model, and it can be seen as a particular type of the global–local shrinkage prior proposed by Tang et al. (2018). In our model, the random effects' variance parameters are area‐specific, and a global parameter γ takes care of the global effects. Our approach's flexibility gives his best performances when large outliers are present: we prove this numerically through several simulated examples. We also apply the proposed model to a real data set regarding the physical activity of the Californian students. We estimate the county‐level proportion of Californian students who obtain a score equal to six to the PFT). Results show that the level of physical activity is significantly related to the county's cultural and economic level, confirming the disparity and heterogeneity among different Californian counties. In real data analysis, our model outperforms its competitors and returns precise and reliable estimates. Here we develop all the necessary detail for implementing the model (the R code is available upon request to the first author).

The FHαS model is more realistic than the Gaussian FH model and has better performance than shrinkage exponential and polynomial‐tailed priors used by Tang et al. (2018) in modeling extreme outliers in the random effects. However, the parameter α governing each random effect's tail‐weight needs to be evaluated by numerical methods and thus intensify the computational effort needed. However, the computational time remains very reasonable, even with hundreds of areas (a few minutes).

The proposed approach can be extended in several directions. The model ignores the spatial correlation among areas that can be included by modifying the random effects' distribution. We are currently working on a multivariate version of the proposed model. Another interesting aspect we do not account for is the measurement error in the covariates: we are studying whether specifying a flexible distribution for the random effect could improve small area predictions even in the presence of covariates measured with error.

## CONFLICT OF INTEREST

The authors have declared no conflict of interest.

### OPEN RESEARCH BADGES

This article has earned an Open Data badge for making publicly available the digitally‐shareable data necessary to reproduce the reported results. The data is available in the Supporting Information section.

This article has earned an open data badge “**Reproducible Research**” for making publicly available the code necessary to reproduce the reported results. The results reported in this article are partially reproducible due to their computational complexity.

## Supporting information

Supporting Information 1

Supporting Information 2
