# Supplementary material for: A new robust Bayesian small area estimation via α‐stable model for estimating the proportion of athletic students in California
Source: Biom J. 2021 May 7;63(6):1309–24. doi: 10.1002/bimj.202000235 (PMC8453931; doi:10.1002/bimj.202000235)
Supplement: Supplementary file 1 — Supporting Information 1 [file BIMJ-63-1309-s002.pdf]

Biometrical Journal **xx** (2020) xx, zzz–zzz / DOI: 10.1002/bimj.200100000

Supplementary materials of the paper A new robust Bayesian small area estimation via α-stable model for estimating the proportion of athletic students in California.

[1] Department of statistics, Faculty of Science, University of Kurdistan, Pasdaran St, Sanandaj,66177-15175, Kurdistan, Iran.
[2] Department of of History, Social Science and Human Studies, University of Salento, Studium 2000, Edificio 5, Via di Valesio, 73100,Lecce, Italy
[3] Department of Statistical Sciences, University of Rome "La Sapienza", piazzale Aldo Moro, 5, 00185, Roma, Italy

Received zzz, revised zzz, accepted zzz

Key words: Small area estimation; Stable distribution; Area-level model; Hierarchical Bayesian model; California FITNESSGRAM.

1 Computational details

Posterior distribution of $\boldsymbol{\beta}$: We write $y_i|v_i,\boldsymbol{\beta},\gamma_1,\alpha \sim N(\boldsymbol{x}_i^T\boldsymbol{\beta}+v_i,\psi_i)$ in the matrix form. Since $\boldsymbol{X}_{m\times p}$ is a full column rank matrix, then has a left inverse. According to the Bayes's rule and Equation (5) since posterior is proportional to the product of the likelihood and prior and using elements that only include $\boldsymbol{\beta}$, we have

$$\begin{aligned}\pi(\boldsymbol{\beta}|\boldsymbol{v},\gamma,\alpha,\boldsymbol{\lambda},\boldsymbol{y}) &\propto \pi(\boldsymbol{y}|\boldsymbol{v},\boldsymbol{\beta},\gamma,\alpha,\boldsymbol{\lambda})\pi(\boldsymbol{\beta})\\ &\propto e^{-\frac{1}{2}\left((\boldsymbol{y}-\boldsymbol{v}-\boldsymbol{X}^T\boldsymbol{\beta})^T\Sigma^{-1}(\boldsymbol{y}-\boldsymbol{v}-\boldsymbol{X}^T\boldsymbol{\beta})\right)}\times e^{-\frac{1}{2}\boldsymbol{\beta}^T\Sigma_\beta^{-1}\boldsymbol{\beta}}\\ &\propto e^{-\frac{1}{2}\left(\boldsymbol{\beta}^T(\boldsymbol{X}\Sigma^{-1}\boldsymbol{X}^T+\Sigma_\beta^{-1})\boldsymbol{\beta}-2\boldsymbol{\beta}^T\boldsymbol{X}\Sigma^{-1}(\boldsymbol{y}-\boldsymbol{v})\right)}\end{aligned}$$

Hence

$$\boldsymbol{\beta}|\boldsymbol{v},\gamma,\alpha,\boldsymbol{\lambda},\boldsymbol{y}\sim N\left((\boldsymbol{X}\Sigma^{-1}\boldsymbol{X}^T+\Sigma_\beta^{-1})^{-1}(\boldsymbol{X}\Sigma^{-1}(\boldsymbol{y}-\boldsymbol{v})),(\boldsymbol{X}\Sigma^{-1}\boldsymbol{X}^T+\Sigma_\beta^{-1})^{-1}\right)$$

where $\Sigma=\mathrm{diag}(\psi_1,\ldots,\psi_m)$.

Posterior distribution of v_i: Furthermore, similar to above paragraph, with the same reasons,

$$\begin{aligned}\pi(v_i|\boldsymbol{\beta},\gamma,\alpha,\boldsymbol{\lambda},\boldsymbol{y}) &\propto \pi(y_i|v_i,\boldsymbol{\beta},\gamma_1,\alpha)\pi(v_i|\boldsymbol{\beta},\gamma_1,\alpha)\\ &\propto e^{\frac{-1}{2\psi_i}(y_i-v_i-\boldsymbol{x}_i^T\boldsymbol{\beta})^2}\times e^{\frac{-1}{2\lambda_i\gamma}v_i^2}\\ &\propto e^{\frac{-1}{2}\left(v_i^2(\frac{1}{\psi_i}+\frac{1}{\lambda_i\gamma})-2(y_i-\boldsymbol{x}_i^T\boldsymbol{\beta})v_i\right)}\\ &= e^{\frac{-1}{2(\frac{1}{\psi_i}+\frac{1}{\lambda_i\gamma})}\left(v_i^2-2\frac{(y_i-\boldsymbol{x}_i^T\boldsymbol{\beta})}{\frac{1}{\psi_i}+\frac{1}{\lambda_i\gamma}}v_i\right)}\end{aligned}$$

by completing the square, we have

$$\pi(v_i|\boldsymbol{\beta},\gamma,\alpha,\boldsymbol{\lambda},\boldsymbol{y}) \propto e^{\frac{-1}{2(\frac{1}{\psi_i}+\frac{1}{\lambda_i\gamma})}\left(v_i-\frac{(y_i-\boldsymbol{x}_i^T\boldsymbol{\beta})}{\frac{1}{\psi_i}+\frac{1}{\lambda_i\gamma}}\right)^2}$$

© 2020 WILEY-VCH Verlag GmbH & Co. KGaA, Weinheim

then

$$v_i|\boldsymbol{\beta}, \gamma, \alpha, \boldsymbol{\lambda}, \boldsymbol{y} \sim N(\delta_i(y_i - \boldsymbol{x}_i^T\boldsymbol{\beta}), \delta_i\psi_i)$$

where $\delta_i = \frac{\lambda_i\gamma}{\lambda_i\gamma+\psi_i}$ and $\gamma = 2\gamma_1^2$.

Posterior distribution of γ: Moreover,

$$\begin{aligned}\pi(\gamma|\boldsymbol{v}, \alpha, \boldsymbol{\lambda}, \boldsymbol{\beta}, \boldsymbol{y}) &\propto \pi(\gamma)\pi(\boldsymbol{v}|\boldsymbol{\beta}, \gamma, \alpha, \boldsymbol{\lambda}) = \pi(\gamma)\prod_{i=1}^{m}\pi(v_i|\boldsymbol{\beta}, \gamma, \alpha, \lambda_i) \\ &\propto (\frac{1}{\gamma})^{a-1}e^{\frac{-b}{\gamma}}(\frac{1}{\gamma})^{\frac{m}{2}}e^{\sum_{i=1}^{m}\frac{-v_i^2}{2\gamma\lambda_i}} = (\frac{1}{\gamma})^{\frac{m}{2}+a-1}e^{\frac{-1}{\gamma}(b+\sum_{i=1}^{m}\frac{v_i^2}{2\lambda_i})}\end{aligned}$$

therefore,

$$\gamma|\boldsymbol{v}, \alpha, \boldsymbol{\lambda}, \boldsymbol{\beta}, \boldsymbol{y} \sim IG\big(a + \frac{m}{2}, b + \sum_{i=1}^{m}\frac{v_i^2}{2\lambda_i}\big)$$

2 Supplementary figures

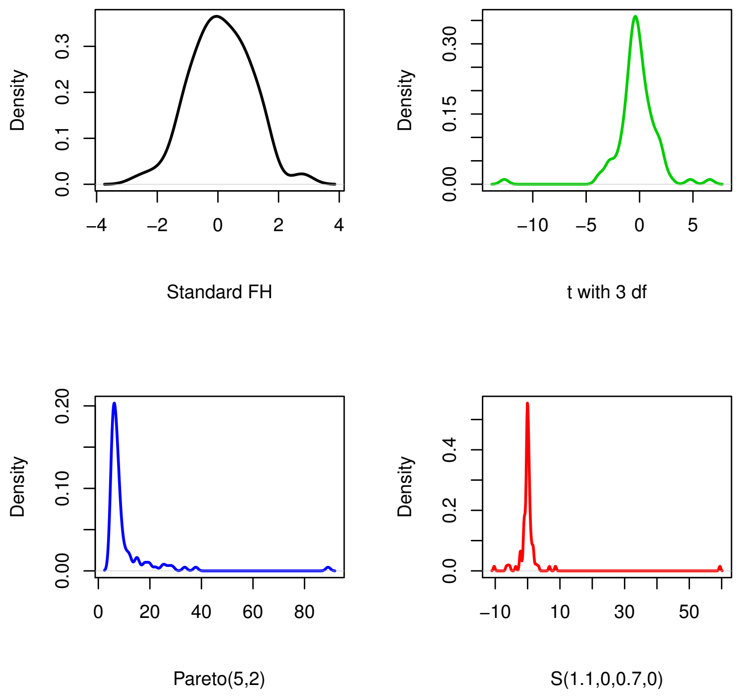

Figure 1 Comparing kernel density plots of the different settings for generating random effects.

© 2020 WILEY-VCH Verlag GmbH & Co. KGaA, Weinheim

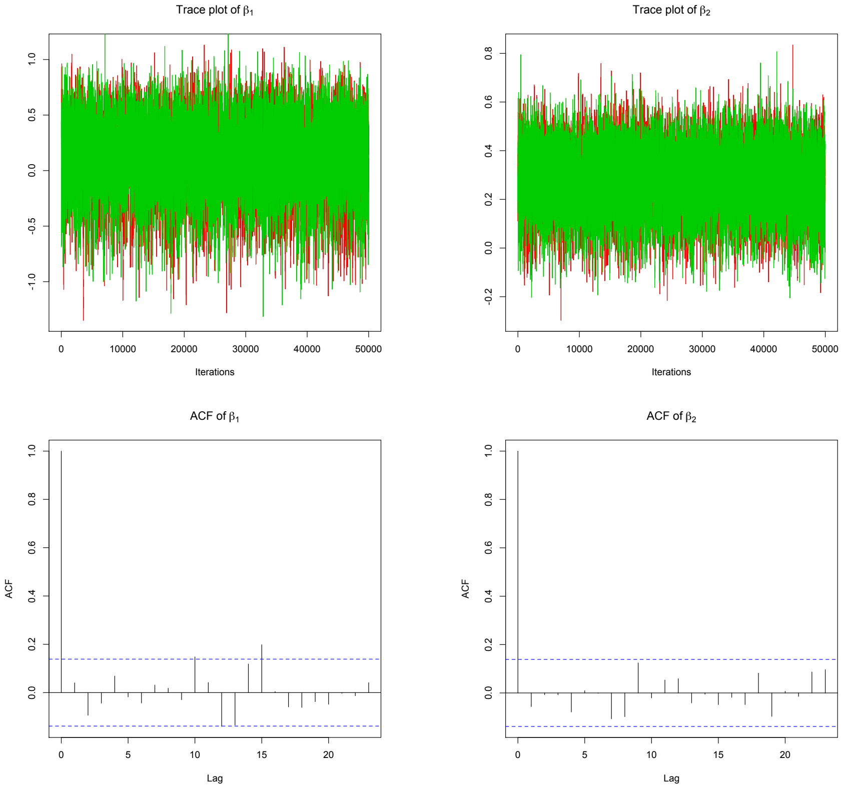

Figure 2 Trace plots (upper panel) and autocorrelation function plots (lower panel) for the parameters β_1 and β_2 of the proposed small area models. Red and green lines in the trace plots correspond to the two MCMC chains.

© 2020 WILEY-VCH Verlag GmbH & Co. KGaA, Weinheim

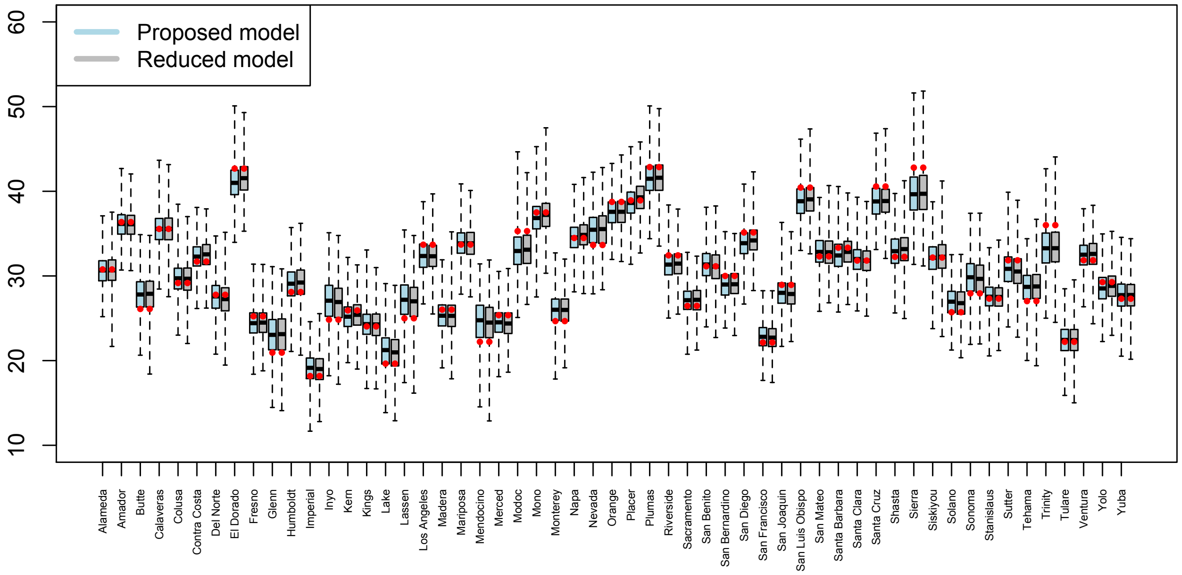

Figure 3 Posterior distribution of the small area means for the proposed model (light blue boxes) and for the proposed model fitted removing the outlying regions of Marin and Tuolumne(grey boxes). Red points correspond to direct estimates.

© 2020 WILEY-VCH Verlag GmbH & Co. KGaA, Weinheim
