# Supplementary material for: A new robust Bayesian small area estimation via α‐stable model for estimating the proportion of athletic students in California
Source: Biom J. 2021 May 7;63(6):1309–24. doi: 10.1002/bimj.202000235 (PMC8453931; doi:10.1002/bimj.202000235)
Supplement: Supplementary file 2 — Supporting Information 2 [file BIMJ-63-1309-s001.zip › Data_Code/README.rtf]

Supplementary information / reproducible research files for the manuscript Title: A new robust Bayesian small area estimation via alpha-stable model for estimating the proportion of athletic students in California.Authors: S. Zarei, S. Arima and G. Jona Lasinio.The code was written/evaluated in R with the following software versions:R version 3.5.1 (2018-07-02)Platform: x86_64-w64-mingw32/x64 (64-bit)Running under: Windows >= 8 x64 (build 9200)Matrix products: defaultattached base packages:[1] parallel  stats     graphics  grDevices utils  methods   base     other attached packages:[1] MASS		GIGrvg		likbstableR		Pareto		codaSimulations as well as real data application were run on MAC	MacBook Pro, 2,9 GHz Intel Core i5 with software version: R version 3.6.0 (2019-04-26)Platform: x86_64-apple-darwin15.6.0 (64-bit)This folder contains the following data and files that can be used to reproduce all analysis and figures of the manuscript.It contains four subfolders containing the following files:./FitData/:An FitData.RData file containing the data analyzed in Section 7 of the paper. The .RData file shows a single data frame, fit, that contains all variables used in the application: the direct estimates, the estimated variances, the county name, the total population, the proportion of population under 18, the proportion of white, black and hispanic and the proportion  of individuals with batchleor degree. All these variables are explained in details in the paper.The R-Code-ScoreDataAnalysis.R: a commented R code for reproducing all the analysis in Section 7. The file Functions.R containing functions necessary to estimate all models and methodologies implemented in Section 7 of the paper../SimulationStudy:The file Functions.R containing functions necessary to generate data according to different simulation scheme described in Section 6 of the paper and all functions for estimating the models.The file Simulations-Scheme.R: a commented R code for reproducing all the analysis in Section 6 of the paper. The code contains also commands for producing all tables in Section 6 of the paper. The user should only select the desired simulation scheme (Scenario 1, Scenario 2, Scenario 3, Scenario 4) as reported in the paper.Notice that the tables in the simulation scenario are obtained without setting a seed. In the file Simulations-Scheme.R we fixa seed and paste the results obtained with the obtained seed. The results, of course, are very similar to those reported inthe paper.Warning: running all simulations and all codes might take long time. If you want to reduce the time, reduce the number of Gibbs simulations.
